# Bis[2,4-dibromo-6-(*n*-propyl­imino­methyl)phenolato-κ^2^ *N*,*O*]cobalt(II)

**DOI:** 10.1107/S1600536810032162

**Published:** 2010-08-18

**Authors:** Chunyan Li, Rui Li, Shufang Zhang

**Affiliations:** a College of Health Science, Wuhan Institute of Physical Education, Wuhan 430079, People’s Republic of China

## Abstract

In the title complex, [Co(C_10_H_10_Br_2_NO)_2_], the Co^II^ atom lies on a twofold rotation axis, the N_2_O_2_ units having distorted tetra­hedral coordination environments comprising two bidentate chelate 2,4-dibromo-6-(*n*-propyl­imino­meth­yl)phenolate Schiff base ligands [Co—N = 1.989 (3) Å, Co—O = 1.924 (2) Å and O/N—Co—O/N = 94.53 (10)–125.40 (15)°]. In the crystal structure, the mol­ecules are linked *via* weak inter­molecular C—H⋯O hydrogen bonds [3.334 (5) Å] and there are also short inversion-related intermolecular Br⋯Br contacts [3.4263 (6) Å]

## Related literature

For related compounds, see: Bermejo *et al.* (1996 ▶); Chen *et al.* (2007 ▶); Li & Wang (2007 ▶); Li *et al.* (2008 ▶); Maneiro *et al.* (2001 ▶); Qiu *et al.* (2007 ▶); Yuan *et al.* (2007 ▶). For standard bond-length values, see: Allen *et al.* (1987 ▶).
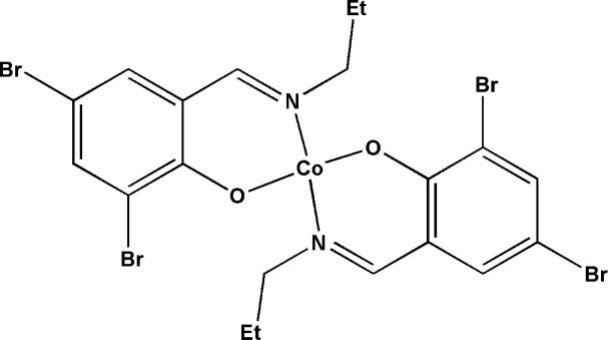


## Experimental

### 

#### Crystal data

[Co(C_10_H_10_Br_2_NO)_2_]
*M* *_r_* = 698.91
Monoclinic, 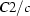

*a* = 24.3684 (10) Å
*b* = 4.8555 (2) Å
*c* = 21.8132 (10) Å
β = 115.523 (4)°
*V* = 2329.08 (19) Å^3^
*Z* = 4
Mo *K*α radiation
μ = 7.62 mm^−1^
*T* = 296 K
0.32 × 0.22 × 0.20 mm

#### Data collection

Bruker SMART CCD area-detector diffractometer
Absorption correction: multi-scan (*SADABS*; Bruker, 2000 ▶) *T*_min_ = 0.097, *T*_max_ = 0.218
6076 measured reflections
2270 independent reflections
1657 reflections with *I* > 2σ(*I*)
*R*_int_ = 0.036

#### Refinement

*R*[*F* ^2^ > 2σ(*F* ^2^)] = 0.030
*wR*(*F* ^2^) = 0.065
*S* = 1.00
2270 reflections
133 parameters
H-atom parameters constrained
Δρ_max_ = 0.41 e Å^−3^
Δρ_min_ = −0.50 e Å^−3^

### 

Data collection: *SMART* (Bruker, 2000 ▶); cell refinement: *SAINT* (Bruker, 2000 ▶); data reduction: *SAINT*; program(s) used to solve structure: *SHELXL97* (Sheldrick, 2008 ▶); program(s) used to refine structure: *SHELXTL* (Sheldrick, 2008 ▶); molecular graphics: *SHELXTL*; software used to prepare material for publication: *SHELXTL*.

## Supplementary Material

Crystal structure: contains datablocks global, I. DOI: 10.1107/S1600536810032162/zs2053sup1.cif

Structure factors: contains datablocks I. DOI: 10.1107/S1600536810032162/zs2053Isup2.hkl

Additional supplementary materials:  crystallographic information; 3D view; checkCIF report

## Figures and Tables

**Fig. 1.**
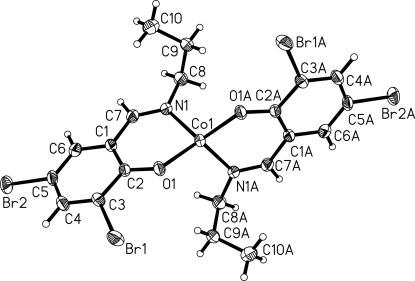
The structure and atom-numbering scheme of the title compound (I), showing 30% probability displacement ellipsoids. The complex has two-fold rotational symmetry, the atoms labeled with the suffix A are related to the primary atoms by the symmetry code -x, y, -z + 3/2.

**Fig. 2.**
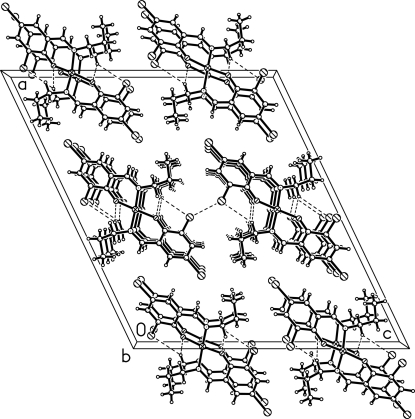
The crystal packing of the title compound (I) viewed along the b axis, linked via intermolecular C—H···O hydrogen bonds (dashed lines).

## supplementary crystallographic information

### Comment

The Lewis base adducts of the 3,5-dibromosalicylidene compounds derived from the condensation of 3,5-dibromosalicylaldehyde with various primary amines are of interest, forming complexes with a large number of transition metals (Chen *et al.*, 2007; Qiu *et al.*, 2007; Maneiro *et al.*, 2001; Bermejo *et al.*, 1996). Recently, mononuclear zinc(II) and nickel(II) compounds of Schiff base ligands derived from the condensation of 3,5-dibromosalicylaldehyde with cyclopropylamine have been structurally characterized (Li & Wang, 2007; Yuan *et al.*, 2007). As an extension of this work, the crystal structure of the title Co^II^ complex, [C_20_H_20_Br_4_CoN_2_O_2_] (I), is reported here.

In (I), the Co^II^ atoms have distorted tetrahedral coordination environments with two bidentate Schiff base ligands, derived from the condensation of 3,5-dibromosalicylaldehyde and *n*-propylamine, acting as chelates through their phenolate O and azomethine N atoms [Co—N 1.989 (3) Å; Co—O 1.924 (2) Å; bond-angle range 94.53 (10)–125.40 (15)°] (Fig. 1). The Co atoms lie on two-fold rotation axes. The C7═N1 bond length of 1.274 (4) Å is somewhat shorter than 1.284 (2) Å observed in the previously reported compound of a Schiff base ligand derived from the condensation of salicylaldehyde with *n*-propylamine (Li *et al.*, 2008). The angle between the two O1—Co1—N1 planes of the molecule is equal to 84.13°. All bond lengths are within normal ranges (Allen *et al.*, 1987). In the crystal structure, the molecules are linked *via* weak intermolecular C—H···O hydrogen bonds and there are also short inversion-related inermolecular Br···Br contacts [3.4263 (6) Å] (Fig. 2).

### Experimental

3,5-Dibromosalicylaldehyde (560 mg, 2 mmol) and *n*-propylamine (118 mg, 2 mmol) were dissolved in methanol (25 ml). The mixture was stirred for 30 min to give an orange solution, which was added to a methanol solution (15 ml) of Co(NO_3_)_2_.6H_2_O (280 mg, 1 mmol). The mixture was stirred for another 20 min at room temperature to give a red solution and then filtered. The filtrate was kept in air for 5 d, forming red blocky crystals. The crystals were isolated, washed three times with distilled water and dried in a vacuum desiccator containing anhydrous CaCl_2_ (yield 68%). Analysis calculated for C_20_H_20_Br_4_CoN_2_O_2_: C 34.37, H 2.88, N 4.01%; found: C 34.17, H 2.90, N 3.99%. IR (KBr, cm^-1^): 3447, 3062, 2966, 2877, 2359, 1619, 1577, 1504, 1434, 1407, 1307, 1212, 1161, 1095, 1040, 865, 838, 749, 705, 604, 466.

### Refinement

All the H atoms were placed in geometrically idealized positions and constrained to ride on their parent atoms, with C—H distances of 0.93–0.97 Å, and with *U*_iso_(H) = 1.2*U*_eq_(carrier) or 1.5*U*_eq_(methyl groups).

### Crystal data

**Table:**

[Co(C_10_H_10_Br_2_NO)_2_]	*F*(000) = 1348
*M**_r_* = 698.91	*D*_x_ = 1.993 Mg m^−^^3^
Monoclinic, *C*2/*c*	Mo *K*α radiation, λ = 0.71073 Å
Hall symbol: -C 2yc	Cell parameters from 1806 reflections
*a* = 24.3684 (10) Å	θ = 3.3–25.5°
*b* = 4.8555 (2) Å	µ = 7.62 mm^−^^1^
*c* = 21.8132 (10) Å	*T* = 296 K
β = 115.523 (4)°	Block, red
*V* = 2329.08 (19) Å^3^	0.32 × 0.22 × 0.20 mm
*Z* = 4	

### Data collection

**Table:**

Bruker SMART CCD area-detector diffractometer	2270 independent reflections
Radiation source: fine-focus sealed tube	1657 reflections with *I* > 2σ(*I*)
graphite	*R*_int_ = 0.036
φ and ω scans	θ_max_ = 26.0°, θ_min_ = 1.9°
Absorption correction: multi-scan (*SADABS*; Bruker, 2000)	*h* = −29→21
*T*_min_ = 0.097, *T*_max_ = 0.218	*k* = −5→5
6076 measured reflections	*l* = −26→26

### Refinement

**Table:**

Refinement on *F*^2^	Primary atom site location: structure-invariant direct methods
Least-squares matrix: full	Secondary atom site location: difference Fourier map
*R*[*F*^2^ > 2σ(*F*^2^)] = 0.030	Hydrogen site location: inferred from neighbouring sites
*wR*(*F*^2^) = 0.065	H-atom parameters constrained
*S* = 1.00	*w* = 1/[σ^2^(*F*_o_^2^) + (0.0251*P*)^2^ + 0.88*P*] where *P* = (*F*_o_^2^ + 2*F*_c_^2^)/3
2270 reflections	(Δ/σ)_max_ < 0.001
133 parameters	Δρ_max_ = 0.41 e Å^−^^3^
0 restraints	Δρ_min_ = −0.50 e Å^−^^3^

### Special details

**Table:**

Geometry. All e.s.d.'s (except the e.s.d. in the dihedral angle between two l.s. planes) are estimated using the full covariance matrix. The cell e.s.d.'s are taken into account individually in the estimation of e.s.d.'s in distances, angles and torsion angles; correlations between e.s.d.'s in cell parameters are only used when they are defined by crystal symmetry. An approximate (isotropic) treatment of cell e.s.d.'s is used for estimating e.s.d.'s involving l.s. planes.
Refinement. Refinement of *F*^2^ against ALL reflections. The weighted *R*-factor *wR* and goodness of fit *S* are based on *F*^2^, conventional *R*-factors *R* are based on *F*, with *F* set to zero for negative *F*^2^. The threshold expression of *F*^2^ > σ(*F*^2^) is used only for calculating *R*-factors(gt) *etc*. and is not relevant to the choice of reflections for refinement. *R*-factors based on *F*^2^ are statistically about twice as large as those based on *F*, and *R*- factors based on ALL data will be even larger.

### Fractional atomic coordinates and isotropic or equivalent isotropic displacement parameters (Å^2^)

**Table:**

	*x*	*y*	*z*	*U*_iso_*/*U*_eq_	
Co1	0.0000	0.31955 (14)	0.7500	0.03770 (18)	
Br1	0.028614 (18)	0.79002 (8)	0.569909 (19)	0.05211 (14)	
Br2	0.232643 (17)	0.11525 (10)	0.62503 (2)	0.06655 (17)	
N1	0.07574 (11)	0.1053 (6)	0.80081 (13)	0.0385 (7)	
O1	0.03028 (9)	0.5013 (5)	0.69236 (11)	0.0413 (6)	
C1	0.11919 (14)	0.2181 (7)	0.72164 (16)	0.0369 (8)	
C2	0.07618 (13)	0.4180 (7)	0.68171 (16)	0.0341 (8)	
C3	0.08482 (14)	0.5258 (7)	0.62579 (16)	0.0368 (8)	
C4	0.13048 (15)	0.4419 (8)	0.60975 (18)	0.0440 (9)	
H4	0.1343	0.5164	0.5725	0.053*	
C5	0.17107 (15)	0.2444 (8)	0.64965 (19)	0.0437 (9)	
C6	0.16661 (15)	0.1351 (8)	0.70498 (18)	0.0468 (9)	
H6	0.1949	0.0056	0.7318	0.056*	
C7	0.11686 (14)	0.0829 (7)	0.77979 (17)	0.0415 (9)	
H7	0.1491	−0.0336	0.8046	0.050*	
C8	0.08417 (17)	−0.0483 (8)	0.86238 (18)	0.0516 (10)	
H8A	0.1148	−0.1894	0.8712	0.062*	
H8B	0.0464	−0.1385	0.8552	0.062*	
C9	0.10364 (16)	0.1417 (9)	0.92354 (18)	0.0559 (11)	
H9A	0.0750	0.2931	0.9127	0.067*	
H9B	0.1023	0.0408	0.9612	0.067*	
C10	0.16709 (18)	0.2569 (10)	0.9451 (2)	0.0746 (14)	
H10A	0.1958	0.1081	0.9567	0.112*	
H10B	0.1772	0.3744	0.9838	0.112*	
H10C	0.1685	0.3610	0.9083	0.112*	

### Atomic displacement parameters (Å^2^)

**Table:**

	*U*^11^	*U*^22^	*U*^33^	*U*^12^	*U*^13^	*U*^23^
Co1	0.0308 (3)	0.0523 (4)	0.0343 (4)	0.000	0.0181 (3)	0.000
Br1	0.0656 (3)	0.0533 (3)	0.0443 (2)	0.0123 (2)	0.03017 (19)	0.01239 (19)
Br2	0.0498 (2)	0.0945 (4)	0.0726 (3)	0.0031 (2)	0.0427 (2)	−0.0139 (3)
N1	0.0333 (15)	0.0490 (19)	0.0339 (16)	−0.0022 (14)	0.0150 (12)	0.0031 (14)
O1	0.0377 (12)	0.0517 (16)	0.0424 (13)	0.0080 (11)	0.0247 (11)	0.0090 (12)
C1	0.0351 (18)	0.044 (2)	0.0357 (19)	−0.0021 (16)	0.0191 (15)	−0.0042 (16)
C2	0.0324 (17)	0.038 (2)	0.0349 (19)	−0.0042 (16)	0.0176 (15)	−0.0035 (16)
C3	0.0448 (19)	0.035 (2)	0.0363 (19)	−0.0020 (16)	0.0230 (16)	−0.0034 (16)
C4	0.050 (2)	0.050 (2)	0.042 (2)	−0.0075 (19)	0.0297 (18)	−0.0053 (18)
C5	0.0372 (19)	0.056 (3)	0.049 (2)	−0.0048 (18)	0.0285 (17)	−0.0104 (19)
C6	0.0334 (19)	0.061 (3)	0.045 (2)	0.0065 (18)	0.0156 (16)	0.0001 (19)
C7	0.0323 (18)	0.049 (2)	0.042 (2)	0.0064 (16)	0.0147 (16)	0.0080 (17)
C8	0.050 (2)	0.060 (3)	0.047 (2)	−0.003 (2)	0.0231 (18)	0.015 (2)
C9	0.046 (2)	0.088 (3)	0.037 (2)	−0.006 (2)	0.0215 (17)	0.010 (2)
C10	0.053 (3)	0.112 (4)	0.051 (3)	−0.016 (3)	0.015 (2)	0.002 (3)

### Geometric parameters (Å, °)

**Table:**

Co1—O1^i^	1.924 (2)	C4—C5	1.383 (5)
Co1—O1	1.924 (2)	C4—H4	0.9300
Co1—N1^i^	1.989 (3)	C5—C6	1.365 (5)
Co1—N1	1.989 (3)	C6—H6	0.9300
Br1—C3	1.889 (3)	C7—H7	0.9300
Br2—C5	1.905 (3)	C8—C9	1.520 (5)
N1—C7	1.274 (4)	C8—H8A	0.9700
N1—C8	1.471 (4)	C8—H8B	0.9700
O1—C2	1.301 (3)	C9—C10	1.516 (5)
C1—C6	1.411 (5)	C9—H9A	0.9700
C1—C2	1.419 (4)	C9—H9B	0.9700
C1—C7	1.451 (4)	C10—H10A	0.9600
C2—C3	1.424 (4)	C10—H10B	0.9600
C3—C4	1.365 (4)	C10—H10C	0.9600
			
O1^i^—Co1—O1	125.40 (15)	C5—C6—C1	120.2 (3)
O1^i^—Co1—N1^i^	94.53 (10)	C5—C6—H6	119.9
O1—Co1—N1^i^	113.63 (10)	C1—C6—H6	119.9
O1^i^—Co1—N1	113.64 (10)	N1—C7—C1	127.5 (3)
O1—Co1—N1	94.53 (10)	N1—C7—H7	116.2
N1^i^—Co1—N1	116.90 (16)	C1—C7—H7	116.2
C7—N1—C8	117.6 (3)	N1—C8—C9	111.2 (3)
C7—N1—Co1	122.0 (2)	N1—C8—H8A	109.4
C8—N1—Co1	120.3 (2)	C9—C8—H8A	109.4
C2—O1—Co1	125.1 (2)	N1—C8—H8B	109.4
C6—C1—C2	120.5 (3)	C9—C8—H8B	109.4
C6—C1—C7	116.1 (3)	H8A—C8—H8B	108.0
C2—C1—C7	123.3 (3)	C10—C9—C8	112.8 (3)
O1—C2—C1	124.6 (3)	C10—C9—H9A	109.0
O1—C2—C3	119.6 (3)	C8—C9—H9A	109.0
C1—C2—C3	115.8 (3)	C10—C9—H9B	109.0
C4—C3—C2	123.2 (3)	C8—C9—H9B	109.0
C4—C3—Br1	118.8 (3)	H9A—C9—H9B	107.8
C2—C3—Br1	117.9 (2)	C9—C10—H10A	109.5
C3—C4—C5	119.1 (3)	C9—C10—H10B	109.5
C3—C4—H4	120.4	H10A—C10—H10B	109.5
C5—C4—H4	120.4	C9—C10—H10C	109.5
C6—C5—C4	121.2 (3)	H10A—C10—H10C	109.5
C6—C5—Br2	119.6 (3)	H10B—C10—H10C	109.5
C4—C5—Br2	119.2 (3)		

Symmetry codes: (i) −*x*, *y*, −*z*+3/2.
